# Efficacy of Remimazolam for Procedural Sedation in American Society of Anesthesiologists (ASA) I to IV Patients Undergoing Colonoscopy: A Systematic Review and Meta-Analysis

**DOI:** 10.7759/cureus.22881

**Published:** 2022-03-06

**Authors:** Ibtehaj Ul-Haque, Taha Gul Shaikh, Syed Hassan Ahmed, Summaiyya Waseem, Nashwa A Qadir, Taha Bin Arif, Shamim Ul Haque

**Affiliations:** 1 Internal Medicine, Dow University of Health Sciences, Karachi, PAK; 2 Internal Medicine, Dr. Ruth KM Pfau Civil Hospital, Karachi, PAK; 3 Department of Anesthesiology, Dalhousie University, Halifax, CAN; 4 Anesthesiology, Saint Martha's Regional Hospital, Antigonish, CAN

**Keywords:** meta-analysis, remimazolam, procedural sedation, midazolam, colonoscopy

## Abstract

Remimazolam is made by combining midazolam and remifentanil as an alternative to conventional sedatives. To evaluate the efficacy of remimazolam for sedation in patients undergoing colonoscopy, we conducted a systematic review and meta-analysis of the available randomized controlled trials (RCTs) comparing remimazolam and midazolam. A search was conducted using PubMed, Cochrane Library, and clinicaltrial.gov from inception till December 26, 2021, for RCTs that investigated the efficacy of remimazolam during the above-mentioned procedure. There was no restriction of language. A quality assessment was performed using the Cochrane Risk-of-Bias tool. The data were pooled, and a meta-analysis was completed. The systemic review was conducted in accordance with the Preferred Reporting Items for Systemic Reviews and Meta-Analysis (PRISMA) guideline statement. Three randomized controlled trials involving 539 patients were included in the meta-analysis. Compared with midazolam during colonoscopy, remimazolam results in reduced need for top-up doses (RR= 3.45, 95% CI=1.07 to 11.14; P=0.04, I^2^=84%). The need for rescue medication was reduced with remimazolam as compared to midazolam (RR=2.42, 95%CI=1.04 to 5.61; P=0.04, I^2^=96%). There was no significant difference observed between the two drugs on completion of colonoscopy and the overall procedural sedation, but the sensitivity analysis favored remimazolam over midazolam for procedural sedation (RR=4.08, 95%CI=2.35 to 7.09; P<0.00001, I^2^=39%). This analysis demonstrates the advantages of remimazolam over other agents and sets a platform for relevant future studies.

## Introduction and background

Introduction

Many gastroenterologists across the world considered sedation an intrinsic part of endoscopy. It aims to reduce the patient’s anxiety and discomfort and decreases the probability of injury during the examination while giving the endoscopist sufficient time to perform a thorough examination [1]. It is defined as a reduction in the level of consciousness through drug-induced mechanisms and ranges from anxiolysis (minimal) to moderate (conscious sedation), deep, and general anesthesia. The most readily used intravenous (IV) sedatives in endoscopic procedures are hypnotic Propofol (an anesthetic alkylphenol) and midazolam (a benzodiazepine), both of which are primarily administered along with fentanyl (an opioid) and have been easily available to physicians since the late 1980s [2-4].

Propofol, a phenol formed by hydroxyl substitution at carbon 2 of 1,3-diisopropyl-benzene, enjoys the reputation of having a short half-life and is reported to have a quick recovery time with minimal incidence of nausea and vomiting [5]. Albeit, it still poses significant limitations. The pain at the injection site is the chief concern for many patients. The American Society of Anesthesiologists (ASA) discourages the use of Propofol in practitioners with insufficient airway skills [3]. The narrow therapeutic index of propofol, because of cardiorespiratory depression, further discourages its use [2].

Midazolam, a benzodiazepine derivative with an imidazole structure, binds to the benzodiazepine receptor at the gamma-aminobutyric acid (GABA)-chloride ionophore complex. This stimulates chloride channels opening, membrane hyperpolarization, and augments the inhibitory effects of GABA. It may also lead to GABA accumulation in the synaptic cleft by interfering with its reuptake [6]. Midazolam, given intravenously, has a lower likelihood of respiratory depression and hypotension. Its wide acceptance by endocrinologists and relatively easier titratability make it suitable for use [7]. It is preferred over propofol because of the availability of its antidote, flumazenil [2-3]. Despite these advantages, there are numerous disadvantages of midazolam, such as extended post-procedural sedation and lengthier hospital stay, especially in patients with renal or hepatic dysfunction due to the elimination half-life of 1.8 to 6.6 hours [2,8]. Many researchers have also reported several adverse effects associated with the use of midazolam. This includes hypotension, respiratory depression, tachycardia, and impairment of cognitive and psychomotor abilities [9].

Remimazolam, a novel ester modified benzodiazepine analog, first received approval in Japan as an intravenous anesthetic in January 2020. It exerts its effect by acting on GABA type A (GABA-A)-chloride ionophore receptor complex like its parent compound midazolam and was approved by the US Food and Drug Administration (FDA) as an alternative to conventional sedatives based on the clinical trials in adults undergoing procedures lasting for 30 minutes or less. This new drug is made by combining midazolam and remifentanil and incorporating carboxylic ester linkage, making it suitable for metabolism [3]. According to the trials, remimazolam has a faster onset of action, good safety profile, and shorter recovery time. It has a terminal half-life of 0.75 hours, shorter than midazolam, which reduces the risk of prolonged post-procedural sedation [8]. Unlike midazolam, it also undergoes organ-independent elimination, making it suitable for patients with renal or hepatic failure [3,10]. However, there is no conclusive evidence on the efficacy of remimazolam available. Therefore, we conducted a systematic review and meta-analysis of the available randomized controlled trials (RCTs) to investigate the efficacy of remimazolam, compared to midazolam, for patients undergoing endoscopy and were categorized into the American Society of Anesthesiologists (ASA) physical status classification I to IV. Anesthesiologists mainly use this classification system to assess patients’ preoperative comorbid conditions and then assign them a class ranging from I-VI. Class I patients are normal healthy patients, whereas classes II and III include patients with mild systemic disease and severe systemic disease, respectively. Patients in class IV have at least one poorly controlled systemic disease (e.g., unstable angina, symptomatic chronic obstructive pulmonary disease, symptomatic chronic heart failure, or hepatorenal failure), which is a constant threat to life. Patients in class V are not expected to survive 24 hours without operation/surgery. ASA class VI patients are declared brain dead [10].

## Review

Material and methods

We followed Cochrane Collaboration guidelines and Preferred Reporting Items for Systematic Reviews and Meta-Analysis (PRISMA) for conducting this systematic review and meta-analysis [11].

Data Sources and Search Strategy

Four authors (IUH, TGS, NAQ, SW) independently used PubMed, the Cochrane Library, and clinicaltrial.gov to conduct a rigorous literature search from inception till December 26, 2021, with no restriction of language. Our search strategy comprised subject headings and free-text terms for “remimazolam”, “midazolam”, “sedation”, “CNS 7056”, and “colonoscopy”, separated by the Boolean operators AND and OR. To achieve comprehensive results, synonyms, related terms, and variant spellings were also engaged. After vigorous research and thorough screening of titles and abstracts, duplicates were removed using EndNote referencing. Moreover, full texts of the retrieved articles were reviewed. Additionally, bibliographies of the recruited articles and relevant reviews were assessed for any relevant data. Authors were also approached via email, in case of any missing data. Following thorough research, studies meeting our predefined inclusion criteria were included.

Inclusion Criteria and Exclusion Criteria

Five authors (IUH, TGS, SHA, NAQ, SW) independently screened all the search results for eligibility. After a full-text review of abstracts, only articles considered relevant by authors were retrieved. Following a full-length review of retrieved articles, their inclusion was confirmed if they met our predefined eligibility criteria. Any disagreements were resolved by discussion with a fifth reviewer (TBA). Studies were included if they met the following inclusion criteria: 1. Randomized controlled trials (RCT), 2. Adult male and female patients (aged ≥18 years), 3. Patients scheduled to undergo a screening or therapeutic colonoscopy procedure, 4. The intervention group having received remimazolam along with fentanyl, 5. Studies reporting midazolam along with fentanyl as an active control, 6. Patients with American Society of Anesthesiologists Physical Status Classification System (ASA) score of I, II, III, or IV, 7. Trials having reported at least one of the desired outcomes. The exclusion criteria were pre-determined as follows: 1. The trial involved an active control group other than midazolam, 2. Trials with a placebo control group only, 3. Dose-finding studies (studies that randomized people into different categories to find a suitable dose of agents with better outcomes), 4. Case reports, letters, reviews, pilot studies, and clinical trials.

Data Extraction and Quality Assessment

Following studies selection, data extraction was undertaken by two independent investigators (TGS, NAQ) and any discrepancies were resolved by discussion with a third reviewer (TBA). The following data were entered into a standard spreadsheet: first author's name, participant characteristics, publication year, study design, sample size, treatment protocols of the intervention and control groups, and outcomes. Clinically, the common outcomes assessed as a measure of sedation efficacy are completion of the procedure, use of alternate/rescue sedatives, patient and clinician satisfaction, pain reported, recall, time to recover, and time to discharge [12]. From the recruited trials, procedure success (represented by composite score) was extracted along with its components: 1. Completion of the endoscopic process, 2. No need for rescue sedative/medication, and 3. No need for more than assigned top-up doses was selected as a measure of efficacy because of the readily available data. Data were available in percentages and were converted to numbers.

The Cochrane Risk of Bias tool (The Nordic Cochrane Centre, Copenhagen, The Cochrane Collaboration) was employed to evaluate each included trial in terms of quality. The following seven domains were assessed: 1. random sequence generation, 2. allocation concealment, 3. selective reporting, 4. blinding of participants/personnel, 5. blinding of outcome assessment, 6. incomplete outcome data, and 7. any other source of bias. To summarize the findings, the risk of bias table was made, grading each domain as high, unclear, or low risk. Each trial was approved by a medical ethics committee according to the respective country's legislation, and all patients or their representatives were informed of the research at the time of inclusion.

Outcome

Our outcome of interest was to assess and systematically evaluate the efficacy of remimazolam and midazolam as sedatives during endoscopic procedures. For this purpose, we used procedure success (composite score), a measure of efficacy. The composite score comprises three outcomes: 1. Completion of endoscopy; 2. Need of rescue sedative (either midazolam or propofol); 3. No more than the assigned top-up dosage, except in one study, Pambianco DJ et al., in which the component “No more than assigned top-up dosage” is missing [4].

Data Analysis

Two independent authors (SHA, SW) performed data analysis using Review Manager (RevMan) 5.4 (The Nordic Cochrane Centre, Copenhagen, The Cochrane Collaboration, 2014) [13]. For dichotomous outcomes, risk ratios (RR) and the corresponding 95% confidence intervals (CIs) were pooled using Mantel-Haenszel random-effects model. In addition, we created forest plots to visualize the results of pooling. The I^2^ statistics, as reported by the forest plots, evaluated statistical heterogeneity across studies, and an I^2^ statistic of >75% was considered significant heterogeneity [14]. We also performed a sensitivity analysis using the “leave-one-out method” to evaluate our outcome.

Results

Literature Search

**Figure 1:**
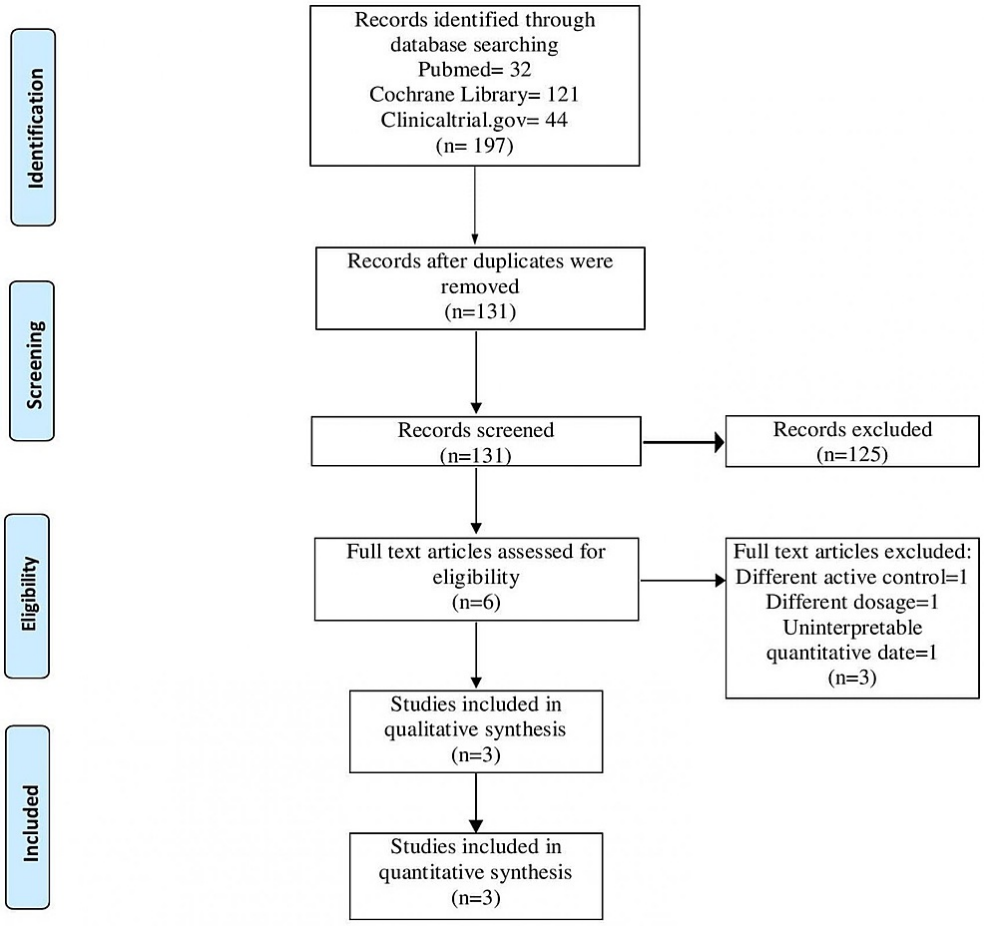
PRISMA flowchart of the systematic review process PRISMA: Preferred Reporting Items for Systematic Reviews and Meta-Analysis

Our initial electronic database searches yielded 197 articles. After the removal of duplicates, the titles and abstracts of 131 articles were screened. A total of six articles were considered relevant and retrieved for full-length review. Following assessment of these full-length articles, ultimately three RCTs were considered eligible for inclusion in this analysis. All the studies included in this meta-analysis fulfill the eligibility criteria as described in materials and methods. The result of our literature search is summarized in the PRISMA flowchart (Figure 1).

Study Characteristics

The included studies assigned a total of 539 patients through the process of randomization. There were 367 patients in the intervention group (remimazolam) and 172 patients in the active-control group (midazolam). The baseline characteristics of the trials are summarized in Table 1. In all three trials, patients of both treatment groups received fentanyl (100 ug in Pambianco et al. [4], 50-75 ug in Rex et al. [7], and 50 ug in Rex et al. [15]) before receiving midazolam or remimazolam.

**Table 1:** Baseline characteristics of the included studies RCT: randomized controlled trial, F: female, M: male, N/A: not available

Study: First author, year	Design	Patients	Procedure	Participant characteristics: intervention group mean (SD) (Age; M/F; BMI)	Participant characteristics: Control group mean (SD) (Age; M/F; BMI)	Intervention Initial dose/top-up dose	Active control initial dose/top-up dose	Outcomes
Pambianco et al., 2016 [4]	RCT	Intervention=40 Control=40	Colonoscopy	N/A	N/A	Remimazolam 5.0/3.0 mg	Midazolam 2.5/1.0 mg	Procedure success, completion of the endoscopic process, no need for rescue sedative/medication
Rex et al., 2018 [7]	RCT	Intervention=296 Control= 102	Colonoscopy	Age=54.4 ± 10.12; M/F=147/149; BMI=28.9 ± 4.72 kg/m2	Age= 55.6 ± 10.15; M/F= 46/56; BMI= 28.8 ± 4.75 kg/m2	Remimazolam 5.0/2.5 mg	Medizolam 1.75/1.0 mg for <60 years; 1.0/0.5 mg >60 years or chronically ill	Procedure success, completion of the endoscopic process, no need for rescue sedative/medication, no more than assigned top-up doses
Rex et al., 2021 [15]	RCT	Intervention=31 Control=30	Colonoscopy	Age=63.1 ± 8.65; M/F=17/14; BMI= 30.9 ± 8.28 kg/m2	Age=61.5 ± 10.60; M/F=14/16; BMI=28.4 ± 6.39 kg/m2	Remimazolam 5.0/2.5 mg	Medizolam 1.75/1.0 mg for <60 years; 1.0/0.5 mg >60 years or chronically ill	Procedure success, completion of the endoscopic process, no need for rescue sedative/medication, no more than assigned top-up doses

Risk of Bias

The risk of bias in individual studies is shown in Table 2. Our included studies reported adequate random sequence generation and low risk for selective reporting, incomplete outcome data, and any other sources of bias. All three included studies reported proper allocation concealment [4,7,15]. All the included studies blinded both participants and outcome assessors to the remimazolam, but in the majority of studies, midazolam was given under open-label use [7,15]. Therefore, these studies were marked high risk for a blinding bias.

**Table 2:** Assessment of methodological quality of the included trials

Study, Year	Random sequence generation	Allocation concealment	Selective reporting	Blinding of participants/personnel	Blinding of outcome assessment	Incomplete outcome data	Other sources of bias
Pambianco et al., 2016 [4]	Low Risk	Low Risk	Low Risk	Low Risk	Low Risk	Low Risk	Low Risk
Rex et al., 2018 [7]	Low Risk	Low Risk	Low Risk	High Risk	High Risk	Low Risk	Low Risk
Rex et al., 2021 [15]	Low Risk	Low Risk	Low Risk	High Risk	High Risk	Low Risk	Low Risk

Results of the Meta-Analysis

**Figure 2:**
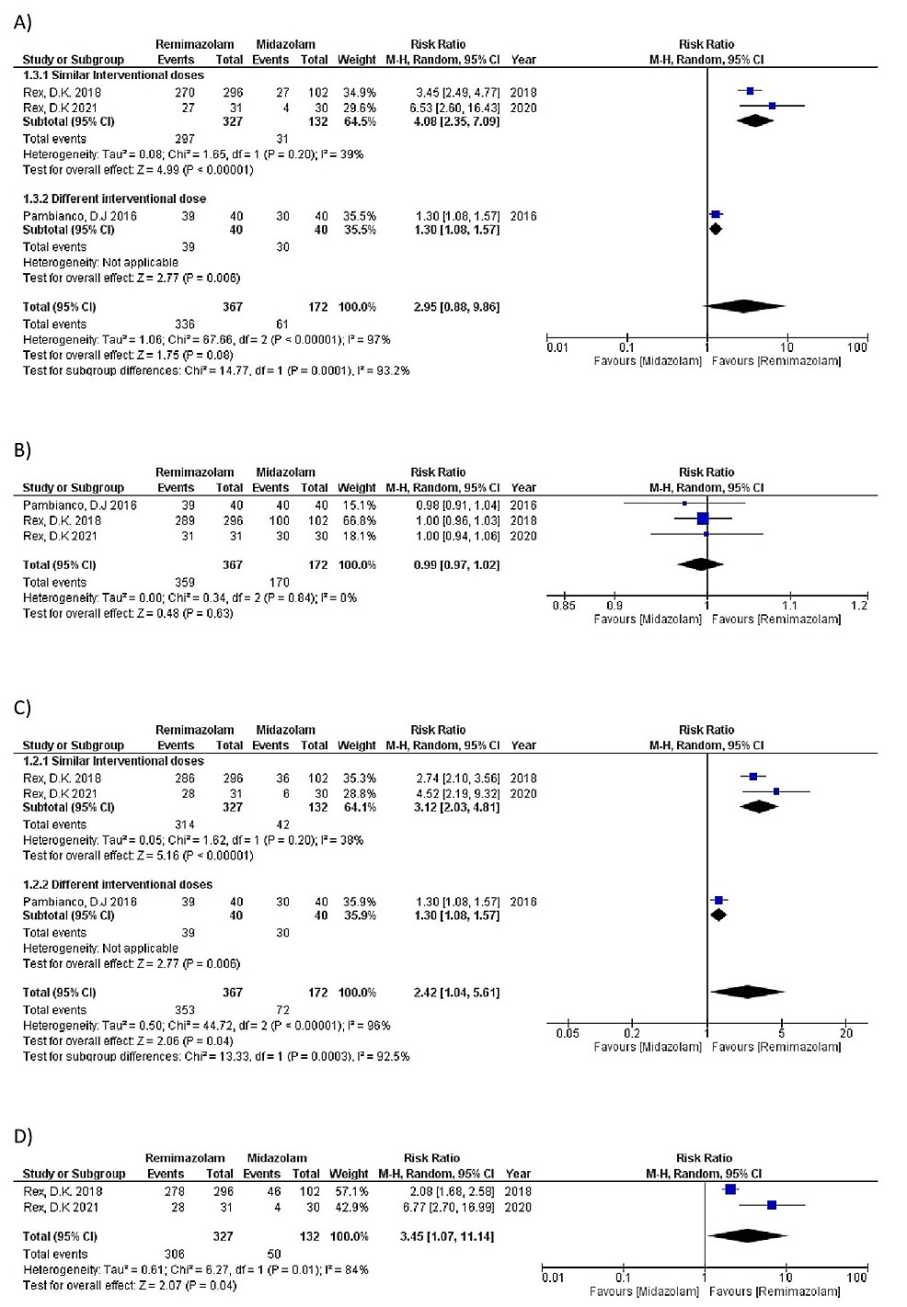
Forest plots of desired outcomes of meta-analysis (A. Procedural success, B. Completion of endoscopy, C. No need for rescue sedative, D. No more than assigned top-up dosage) CI: confidence interval; M-H: Mantel-Haenszel; df: degrees of freedom Rex, DK 2018 [7]; Rex, DK 2021 [15]; Pambianco, DJ 2016 [4]

A. Procedural success: All the selected studies reported procedural success as a composite score (remimazolam: 367 patients, 336 events; midazolam: 172 patients, 61 events). The pooled results revealed a statistically non-significant association of remimazolam with procedural success compared to midazolam (RR=2.95, 95% CI=0.88 to 9.86; P=0.08, I^2^=97%) as shown in Figure 2. However, sensitivity analysis, by removing the study with different drug doses [4], strongly favored remimazolam (RR=4.08, 95%CI=2.35 to 7.09; P<0.00001, I^2^=39%). The result of this outcome is demonstrated in Figure 2A.

B. Completion of endoscopy (colonoscopy): Data for this outcome were available in all the included studies (remimazolam: 367 patients, 359 events; midazolam: 172 patients, 170 events). There was statistically no significant relationship observed between the two drugs (RR=0.99, 95%CI=0.97 to 1.02; P=0.63, I^2^=0%) as shown in Figure 2B. Similarly, no significant association was observed by performing sensitivity analysis.

C. No need for rescue sedative: All the included studies reported this outcome (remimazolam: 367 patients, 353 events; midazolam: 172 patients, 72 events). Using remimazolam significantly reduced the need for rescue medication compared to midazolam (RR=2.42, 95%CI= 1.04 to 5.61; P=0.04, I^2^=96%) as shown in Figure 2C. Sensitivity analysis, by removing the study with different doses [4], also favored remimazolam over midazolam (RR=3.12, 95%CI=2.03 to 4.81; P<0.0001, I^2^=38%). 

D. No more than assigned top-up dosage: Two out of three studies provided the raw data for this outcome (remimazolam: 327 patients, 306 events; midazolam: 132 patients, 50 events). A significant difference was observed in favor of remimazolam (RR= 3.45, 95%CI= 1.07 to 11.14; P=0.04, I^2^=84%), as shown in Figure 2D.

Discussion

Our analysis of a total of 539 patients provides evidence regarding the higher efficacy of remimazolam over midazolam in patients undergoing colonoscopy. Although two recent reviews have highlighted that remimazolam has an edge over midazolam in terms of efficacy and better safety profile, to the best of our knowledge, this study is the first to pool the results of individual trials and statistically compare the two drugs involving the patients undergoing colonoscopy [2-3]. Additionally, in this study, only dose-specific trials of remimazolam were included i.e., studies involving up to 5 mg of the loading dose, since it is reported to have higher efficacy and safety profile with a top-up dose of 3.0 mg [4]. While we were drafting our article, we found two recent systematic reviews and meta-analyses evaluating the efficacy and safety of remimazolam in patients undergoing endoscopy. Both the studies have highlighted that remimazolam has higher efficacy in terms of better sedation and procedural success, which is consistent with our analysis [16,17]. Both the studies have included dose-finding and dose-specific trials in their analysis, whereas our analysis only includes dose-specific trials of remimazolam i.e., up to 5.0 mg of a loading dose of remimazolam. This is because there is no advantage in dosing healthy individuals by weight rather than a specific dose [18]. Moreover, our analysis is solely focused on colonoscopy, unlike the two meta-analyses, which have included bronchoscopy and upper gastrointestinal (GI) endoscopy too. Moreover, one study mentioned that since their analysis had both fixed-dose studies and dose-finding studies, the result might not be accurate in terms of dose to be used for the endoscopic procedure [17]. Similarly, the patients from class V and VI were not included because of the paucity of the data and were not deemed suitable for the procedure, respectively. The patients categorized into class IV, because they had at least one poorly controlled disease or were at end-stage, are at the risk of developing unstable angina, hepatorenal failure, and even death [10]. This might have impacted the results.

Our analysis highlights that the use of remimazolam significantly reduced the need for top-up doses and the need for rescue sedatives. Additionally, the subgroup analysis based on different loading and top-up doses of remimazolam and midazolam also favored remimazolam for higher procedural success and reduced need for rescue sedative. These results are consistent with the previous trials [4,15]. However, the result for another component of the procedural success, i.e., completion of endoscopy, was not significant.

All the trials in our intervention group (remimazolam) followed the FDA-approved guidelines. According to the guideline, a loading dose of 5.0 mg and for people falling under ASA III classification, a loading dose of 2.5 mg to 5.0 mg is permissible. Similarly, a top-up dose of 2.5 mg and for people in the ASA III classification, a top-up dose ranging from 1.25 mg to 2.5 mg is approved [19]. One exception is the usage of 3.0 mg as a top-up dose in the study by Pambianco et al. [4]. Similarly, the dosage in the active control group was in accordance with the FDA guidelines. The guidelines allow for the maximum loading dose of 2.5 mg in healthy individuals and up to 3.5 mg in chronically ill patients [20].

The potential explanation for the higher efficacy of remimazolam is in its underlying mechanism of action. It has a high affinity and selectivity for the benzodiazepine site on the GABA receptors [21-22]. Furthermore, the metabolite of remimazolam has a lower affinity for the benzodiazepine receptors than the metabolite of midazolam, α-hydromidazolam [23]. The addition of a carboxylic ester in the benzodiazepines makes it receptive to tissue esterases (CES), resulting in a pharmacologically inactive metabolite, CNS-7054. This explains the short life of remimazolam compared to midazolam. It has a quick clearance rate, minimum variability among individuals, and a small volume of distribution [24]. Moreover, the organ-independent elimination of remimazolam makes it suitable for patients with hepatic or liver dysfunction [3]. This property is of great importance and makes remimazolam suitable for use in a wider population. Even though one trial included in this analysis highlights that remimazolam, for endoscopy, is independent of ASA classification, there is insufficient data for statistical analysis [15]. Our analysis highlighted that dose-specific medication has a greater chance of success as highlighted in our outcome i.e., procedural sedation. The results were initially insignificant. However, upon sensitivity analysis depending on the top-up used, the results were significant when 5.0/2.5mg of remimazolam was used. This analysis highlights that in the future, more specific drug doses should be considered for trials. We found little variation in the outcome “completion of the endoscopic procedure (colonoscopy)” because there were few instances where the treatment failure was declared.

The most common adverse effects usually reported after the use of remimazolam are hypertension, hypotension, bradycardia, decreased respiratory rate, and hypoxia. However, no statistically significant difference was observed between both the intervention groups for the adverse effects as reported in the previous two meta-analyses except for the incidence of hypotension, for which remimazolam had a better outcome [16-17]. Other minor side effects include nausea and vomiting. Despite these adverse effects, the benefits of remimazolam outweigh the risk. Our systematic research revealed a paucity of trials on the use of remimazolam for colonoscopy. As more trials are carried out on this drug, we will have a clearer idea of the efficacy of remimazolam.

This study carries some limitations that need to be highlighted so that in the future, more specific trials, in terms of population and dosages, are carried out. First, this meta-analysis was carried out assuming that all the baseline characteristics of the patients included in the trials were similar. Inconsistency in baseline characteristics and different initial and top-up doses of fentanyl in both treatment groups could have contributed to high clinical heterogeneity. Second, the trials in our analyses included patients of different ASA classification, which also could have contributed to the already high heterogeneity. Furthermore, the sensitivity analysis was not carried out to find the effect of other confounding factors, such as age, body mass index (BMI), and diseases, including respiratory disorders, diabetes, and hypertension due to the unavailability of required data. Third, all the trials were carried out in the United States of America, and hence, these results may be marked by significant variations across different demographics. Additionally, all the included studies were sponsored by PAION AG (Aachen, Germany), a pharmaceutical company, leaving room for industry sponsorship bias. Lastly, the trials only encompassed the intravenous administration of remimazolam. Even though a trial on the intranasal route was conducted, and despite that, the clinical outcomes were positive, practical use of this route is not possible due to excruciating discomfort during delivery [25]. Similarly, a study by Pesic et al. reported the oral bioavailability of remimazolam to be only 1.2-2.2%, which renders it useless through the said route [26]. We did not investigate the publication bias due to limited studies. Going forward, more well-powered clinical trials should be conducted for more accurate results.

## Conclusions

Several countries, including Europe and the United States of America, are conducting trials on the usage of remimazolam in procedural sedation. The trials conducted so far demonstrate that it has the potential to replace midazolam for procedural sedation. There is a paucity of data regarding the use of this drug in different endoscopic types, i.e., bronchoscopy, upper GI endoscopy, and colonoscopy, but this analysis sets the platform for future trials to test this drug in different demographic settings. Similarly, there is potential to carry out more trials in obese populations, ICU settings, and vulnerable populations (e.g., with respiratory disorders). Moreover, more trials for each mentioned ASA classification should be carried out to get better results. The overall procedural success of remimazolam is striking. However, certain aspects of this drug need careful examination, such as cost-effectiveness and safety profile, before its wide acceptance and usage.
